# Morphometric imaging biomarker identifies Alzheimer’s disease even among mixed dementia patients

**DOI:** 10.1038/s41598-022-21796-y

**Published:** 2022-11-01

**Authors:** Florin V. Chirila, Guang Xu, Dan Fontaine, Grant Kern, Tapan K. Khan, Jason Brandt, Yoshihiro Konishi, Gerhard Nebe-von-Caron, Charles L. White, Daniel L. Alkon

**Affiliations:** 1 Synaps Dx, 12358 Parklawn Drive, Rockville, MD 20852 USA; 2 Spot Dx, 895 Vandalia Rd., Morgantown, WV 26501 USA; 3 Johns Hopkins Hospital Psychiatry, 600 N Wolfe St # 384, Baltimore, MD 21287 USA; 4 National Hospital Organization Tottori Medical Center Tottori, 876 Mitsu, Tottori, 689-0203 Japan; 5 Mologic, Bedford Technology Park, Thurleigh, Bedfordshire, MK44 2YA UK; 6 Department of Pathology, The University of Texas Southwestern Medical Center, 5323 Harry Hines Blvd, Dallas, TX 75390-9073 USA

**Keywords:** Biotechnology, Neuroscience, Biomarkers, Neurology

## Abstract

A definitive diagnosis of Alzheimer’s disease (AD), even in the presence of co-morbid neuropathology (occurring in > 50% of AD cases), is a significant unmet medical need that has obstructed the discovery of effective AD therapeutics. An AD-biomarker, the Morphometric Imaging (MI) assay on cultured skin fibroblasts, was used in a double-blind, allcomers (ages 55–90) trial of 3 patient cohorts: AD dementia patients, N = 25, all autopsy confirmed, non-AD dementia patients, N = 21—all autopsy or genetically confirmed; and non-demented control (AHC) patients N = 27. Fibroblasts cells isolated from 3-mm skin punch biopsies were cultured on a 3-D Matrigel matrix with movement dynamics quantified by image analysis**.** From counts of all aggregates (N) in a pre-defined field image and measures of the average area (A) of aggregates per image, the number-to-area ratios in a natural logarithmic form Ln(A/N) were determined for all patient samples. AD cell lines formed fewer large aggregates (cells clustered together) than non-AD or AHC cell lines**.** The cut-off value of Ln(A/N) = 6.98 was determined from the biomarker values of non-demented apparently healthy control (AHC) cases. Unequivocal validation by autopsy, genetics, and/or dementia criteria was possible for all 73 patient samples. The samples were collected from multiple centers—four US centers and one center in Japan. The study found no effect of center-to-center variation in fibroblast isolation, cell growth, or cell aggregation values (Ln(A/N)). The autopsy-confirmed MI Biomarker distinguished AD from non-AD dementia (non-ADD) patients and correctly diagnosed AD even in the presence of other co-morbid pathologies at autopsy (True Positive = 25, False Negative = 0, False Positive = 0, True Negative = 21, and Accuracy = 100%. Sensitivity and specificity were calculated as 100% (95% CI = 84 to 100.00%). From these findings, the MI assay appears to detect AD with great accuracy—even with abundant co-morbidity.

## Introduction

**Figure 1:**
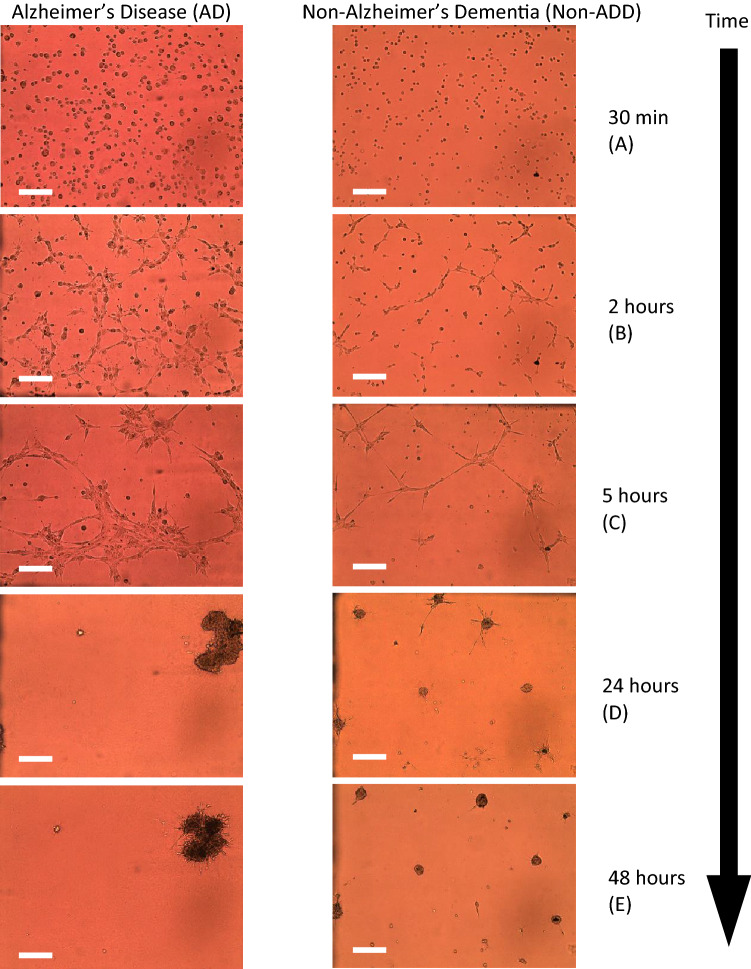
Time course study of cultured skin fibroblasts obtained from Alzheimer’s and non-Alzheimer’s disease (AD/non-ADD) patients on a 3-D Matrigel matrix. AD and non-ADD dementia cells are cultured, and images are taken at 30 min (**A**), 2-h (**B**), 5-h (**C**), 24-h (**D**), and 48-h (**E**) intervals. All images are taken in 10X objective. The scale bar is 250 µm.

**Figure 2:**
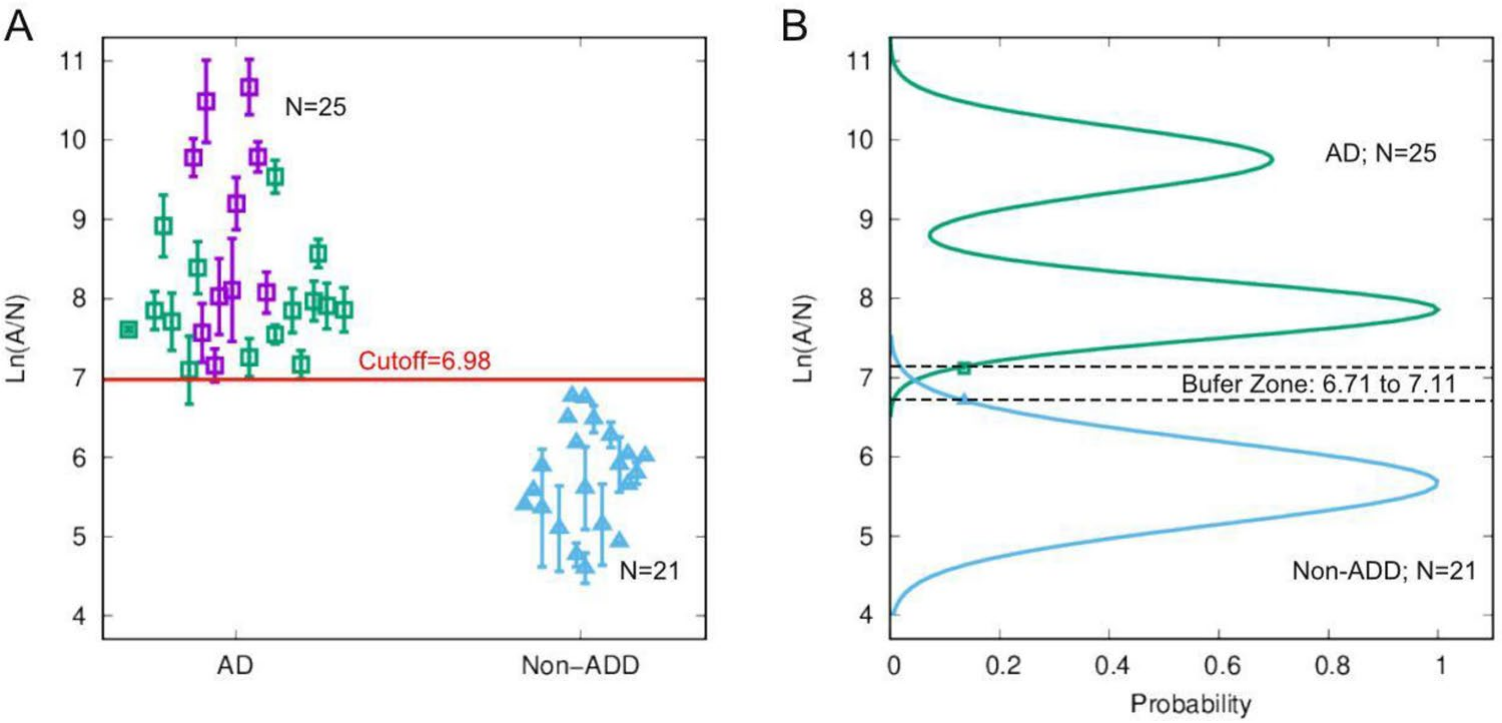
(**A**) Ln(A/N) Morphology Index (MI) values in human fibroblast cultures on Matrigel matrix. The MI index was plotted for cells of patients from two different categories: (i) AD (purple squares), (ii) comorbid AD (AD with other non-AD pathology (mixed diagnosis of AD, Parkinson’s disease, and Lewy body disease, frontotemporal dementia, etc.-green squares), (iii) non-AD dementia (non-ADD, i.e., Parkinson’s disease FTD, LBD—blue triangles), all cell lines from patients confirmed by autopsy. This method accurately diagnosed AD patients distinguishing them from non-ADD patients. (**B**) Probability Gaussian distributions for the AD (green) and non-ADD populations (blue) as a function of Ln(A/N). The dashed black lines represent the buffer zone boundaries determined at the level of four standard deviations for each Gaussian distribution (see methods section). The small peak in the AD distribution is due to high passage (> 10) cell lines.

Alzheimer’s disease (AD) has remained refractory to therapeutics that treat the underlying condition^1^. Over the past two decades, clinical trials that tested potential AD therapeutics often targeted patients without a definitive neuropathological diagnosis for AD dementia. Moreover, clinical diagnoses are highly inaccurate, particularly during the first 4 to 5 years of disease duration^2^. This urgent unmet medical need for a highly accurate, easily accessible AD biomarker has motivated the development of several candidate AD tests. These included MRI and PET imaging of amyloid plaques^3–5^, CSF and plasma measures of soluble amyloid and tau, and blood levels of tau^6–9^. While some of these blood biomarkers showed high sensitivity, the specificity was often lower when validated with autopsy pathology. The specific distinction of AD from non-ADD was often not supported by the autopsy pathologies^10–12^. For example, one study^10^ demonstrated a significant overlap of AD and non-ADD values of plasma p-tau 217 (Palmqvist S, Janelidze S, Quiroz YT, et al. Discriminative Accuracy of Plasma Phospho-tau217 for Alzheimer Disease vs. Other Neurodegenerative Disorders. JAMA. 2020; 324(8):772–781. https://doi.org/10.1001/jama.2020.12134): see eFigure 2, eTable 10 (specificity of 79% for plasma p-tau 217), Figure 2A (significant AD overlap vs. non-ADD values of plasma p-tau 217), Figure 1d (significant AD overlap vs. non-ADD values of plasma p-tau 181), Table 2 (significant AD overlap vs. non-ADD values of Plasma p-tau 181- specificities of 67.2% between AD and Frontotemporal Lobar dementia (FTLD) + TDP + FTLD-tau, and specificity of 63.5% between autopsy-confirmed AD and FTLD-tau, and specificity of 62.6% between autopsy-confirmed AD and FTLD + mutation carriers^10^.

A second study^11^ demonstrated 79% sensitivity and specificity (Janelidze S, Berron D, Smith R, et al. Associations of Plasma Phospho-Tau217 Levels with Tau Positron Emission Tomography in Early Alzheimer Disease. JAMA Neurol. 2021;78(2):149–156. https://doi.org/10.1001/jamaneurol.2020.4201).

A third study^12^ had several pathology-confirmed AD’s (15) and FTLD’s (68). However, when considering the “frequent” category in eFigure 5, the specificity for p-tau 181 was 71%, with a 95% confidence interval between 29 and 96%. Similarly, for p-tau 217, the specificity was 67%, with a 95% confidence interval between 22 and 96%) (Thijssen EH, La Joie R, Strom A, et al. Advancing Research and Treatment for Frontotemporal Lobar Degeneration investigators. Plasma phosphorylated tau 217 and phosphorylated tau 181 as biomarkers in AD and FLTD: a retrospective diagnostic performance study. Lancet Neurol. 2021; 20(9):739–752. https://doi.org/10.1016/S1474-4422(21)00214-3).

As a result, those biomarkers would not be effective in completely differentiating AD from Parkinson’s disease dementia, Lewy Body dementia, Frontotemporal dementia, dementia due to Cortico-basal syndrome, dementia due to Progressive supranuclear palsy, a behavioral variant of Frontotemporal dementia (bvFTD), primary progressive aphasia, and FTLD. A recent review estimated that blood plasma Aβ42/ Aβ40 has 62–72% accuracy for the differential diagnosis of AD vs. non-ADD^13^. By way of contrast, the AD-Index Biomarker (developed for skin fibroblasts as used in the Morphology Imaging Assay presented here) based on 68 autopsies showed high sensitivity and specificity (> 95%)^2,14^. The AD-Index Biomarker in those studies used a 3-mm skin biopsy to isolate fibroblasts. Cultured fibroblasts were incubated to 80–90% confluence. An inflammatory agonist (a small nano-peptide, bradykinin, that induces Erk1 and Erk2 phosphorylation in fibroblasts) stimulated the skin cells. Quantitative imaging of the phosphorylated Erk1 and Erk2 was then used to identify and differentiate AD from Non-AD dementia and age-matched control (AC) specimens. In this same earlier skin fibroblast biomarker study, while the AD-Index Biomarker was highly accurate (> 95%), clinical diagnoses showed < 60% accuracy during the first four years when referenced to blinded neuropathological diagnoses^2^. Furthermore, this skin fibroblast AD biomarker, but no other to date, showed high diagnostic accuracy for cases in which the autopsy pathology included AD together with additional pathologies (i.e., co-morbidities) due to other forms of neurodegeneration, such as multi-infarct dementia, Parkinson’s disease, Lewy-Body dementia, and frontotemporal dementia. Nevertheless, it is now well-known that many autopsies (> 50%) have demonstrated this co-morbidity, which makes the AD diagnosis even more challenging^15–23^.

Specific dysfunctions in skin fibroblasts from AD patients with diagnostic potential have included abnormalities in K + channels^24^, PKC isozymes^25–27^, MAP Kinase ERK1/2^2,14^, folate binding^28^, and cholesterol processing^29^. Previously, we presented a skin AD biomarker based on skin fibroblast aggregation^30,31^ that distinguishes AD from non-ADD and non-demented control cases.

Several clinical-pathological studies have shown that the degree of cognitive dysfunction measured clinically for AD-demented patients closely correlates with synaptic loss, but does not closely correlate with the abundance of amyloid plaques and neurofibrillary tangles in autopsy brain samples^32,33^. Biomarkers that could relate to the formation of networks among neurons that are functionally connected via synapses were developed to identify patients with AD dementia in the presence of amyloid plaques, neurofibrillary tangles, and synaptic loss. To date, these biomarkers have included the levels of PKC-epsilon^25^ that regulate synaptic growth and neuronal apoptosis, downstream molecular targets of PKC-epsilon, such as ERK1 and ERK2^14^, and, as here analyzed, the movement and behavior of skin fibroblasts cells to positions of proximity and contact.

In previously published articles^30,31^ on the Morphometric Imaging (MI) AD-Biomarker assay presented fibroblasts from the cultured skin specimens were stimulated with an extracellular matrix composed of an array of macromolecules (3-D Matrigel), forming networks that were dysregulated in AD^30,31^. Fibroblast aggregates or “networks” were differentially formed for age-matched control and non-AD dementia cells compared to AD skin fibroblast cells. Based on differences in the rate and extent of network formation, a highly accurate diagnostic biomarker of AD was reported that was validated with autopsy pathologic hallmarks of AD, namely amyloid plaques and neurofibrillary tangles^31^. This biomarker accurately diagnosed AD patients and distinguished them from AC and non-AD dementia specimens^30,31^. Here, with additional skin fibroblast samples, we examined the accuracy of the MI Biomarker Assay when AD pathology occurred together with other co-morbid neurodegenerative pathologies. The results support the diagnostic accuracy for identifying AD even in the presence of co-morbid neuro-degenerative pathologies demonstrated at autopsy. The samples presented were based on prospective cohorts identified without formal registration in a clinical trial. This report does not refer to a registered clinical trial. It is a prospective cohort study of whether or not Morphometric Imaging (MI) biomarker identifies AD, even among mixed dementia patients – using previously unreported samples collected in a study conducted in collaboration with the Alere Diagnostics Company (now a part of Abbott).

## Results

The samples were collected from the Coriell Institute, NJ, multiple centers—four US and one in Japan. Among the 73 samples utilized in all calculations, 49% were from the Coriell Institute, NJ, 19% were from the Copper Ridge Institute, MD, and the rest of 32% were from all other sources. The study found no effect of center-to-center variation in fibroblast isolation, cell growth (Supplementary Documents Table S1), and cell aggregation values (Ln(A/N)) (Tables 1, 2).

**Table 1:** Patient population: Autopsy-validated fibroblasts-based peripheral diagnosis of Alzheimer’s disease and the presence of co-morbidity (N = 25).

Sample ID/Source	Age	Gender	Method	Disease duration (Yrs)	Ln(A/N)	MI diagnosis	History and risk factors	Autopsy findings
538 UTSW	79	M	Autopsy	N/A	8.39	AD	N/A	Pure Alzheimer’s disease; comorbidity = 0
539 UTSW	77	M	Autopsy	N/A	7.1	AD	N/A	Pure Alzheimer’s disease; comorbidity = 0
557 UTSW	78	M	Autopsy	N/A	7.55	AD	N/A	Alzheimer’s disease, lewy body dementia; comorbidity = 1
AG05770 Coriell	70	M	Autopsy	7.5	8.11	AD	None	Pure Alzheimer’s disease; comorbidity = 0
AG08245 Coriell	75	M	Autopsy	7	9.78	AD	None	Alzheimer’s disease, Parkinson’s disease, lewy body dementia; comorbidity = 2
AG08527 Coriell	61	M	Autopsy	N/A	7.57	AD	None	Alzheimer’s disease, Parkinson’s disease; comorbidity = 1
AG06840 Coriell	56	M	Genetic	4	9.2	AD	None	Pure Alzheimer’s disease; comorbidity = 0
AG04159 Coriell	52	F	Genetic	8	10.67	AD	Complex partial seizures	Pure Alzheimer’s disease; comorbidity = 0
AG06844 Coriell	59	M	Genetic	11	9.79	AD	Progressive memory impairment, diffuse slowing EEG	Pure Alzheimer’s disease; comorbidity = 0
AG10788 Coriell	87	Unknown	Genetic	17	8.08	AD	None	Pure Alzheimer’s disease; comorbidity = 0
AG06869 Coriell	60	F	Autopsy	1	8.03	AD	Visuospatial disorientation, dyscalculia, diffuse slowing EEG	Pure Alzheimer’s disease; comorbidity = 0
AG11368 Coriell	77	M	Autopsy	N/A	7.16	AD	None	Pure Alzheimer’s disease; comorbidity = 0
AG05810 Coriell	79	F	Autopsy	N/A	10.49	AD	None	Pure Alzheimer’s disease; comorbidity = 0
550 UTSW	72	M	Autopsy	N/A	9.54	AD	None	Pure Alzheimer’s disease; comorbidity = 0
588 CRI	78	M	Autopsy	N/A	7.85	AD	Cerebrovascular accident, hypertension, benign prostatic hypertrophy, renal insufficiency, hemorrhoids, glaucoma	Alzheimer’s Disease, Lewy Body Dementia, Lateral Ventricle Hydrocephalus; Comorbidity = 2
658 CRI	88	F	Autopsy	N/A	7.17	AD	Coronary artery disease, hypertension, anemia, glaucoma, perioral tremor, hysterectomy	Alzheimer’s Disease, Frontotemporal Lobar Degeneration with TDP-43-Positive Inclusions; Comorbidity = 1
568 CRI	87	F	Autopsy	N/A	8.92	AD	Hypercholesterolemia, coronary artery disease, hypertension, depression, serious head injury	Alzheimer’s Disease, Vascular Dementia, Multiple Remote Infarcts; Comorbidity = 2
575 CRI	55	F	Autopsy	N/A	8.92	AD	Osteoporosis, migraines, vitamin D deficiency, seizure disorder	Alzheimer’s Disease, Frontotemporal Dementia, Marked Hydrocephalus; Comorbidity = 2
589 CRI	80	F	Autopsy	N/A	7.97	AD	Vascular dementia, hypertension, hyperlipidemia, hyperhomocysteinemia, hyperthyroidism, anemia, depression	Alzheimer’s disease, vascular dementia, left occipital lobe infarct, multiple remote microinfarcts, lewy body disease; comorbidity = 4
578 CRI	70	M	Autopsy	N/A	7.91	AD	Early-onset dementia, seizures, diverticulosis, attention deficit disorder	Alzheimer’s disease, frontotemporal lobar degeneration with TDP-43-positive inclusions, lewy body disease, amygdalar pattern; comorbidity = 5
563 CRI	81	F	Autopsy	N/A	7.86	AD	Anxiety disorder, colon cancer, severe headaches, intracerebral hemorrhage	Alzheimer’s disease, vascular dementia, right medial frontal-parietal cortex hemorrhage, remote right lateral prefrontal cortex hemorrhage, remote hemorrhagic infarct, multiple microinfarcts
652 CRI	86	F	Autopsy	N/A	7.85	AD	Diabetes, renal insufficiency, coronary artery disease, primary pulmonary hypertension, edema, atrial fibrillation, hyperlipidemia, hypothyroidism, anemia, osteopenia	Alzheimer’s disease, vascular dementia, multiple remote infarcts; comorbidity = 2
599 WHM	79	M	Autopsy	N/A	7.71	AD	Depression	Alzheimer’s disease, hydrocephalus; comorbidity = 1
587 CRI	85	F	Autopsy	N/A	7.61	AD	Labile hypertension, hypercholesterolemia, osteoporosis, colon adenocarcinoma, stroke	Alzheimer’s disease, lewy body disease -neocortical type with alpha-synuclein-immunoreactive astrocytosis; comorbidity = 1
590 CRI	90	F	Autopsy	N/A	7.26	AD	Labile hypertension, hypercholesterolemia, hypothyroidism, osteoporosis	Alzheimer’s disease, multiple cortical infarcts, moderate hydrocephalus; comorbidity = 2

**Table 2:** Autopsy/genetically validated skin fibroblasts-based peripheral diagnosis of non-Alzheimer’s disease dementia (N = 21).

Sample ID	Age	Gender	Non-ADD diagnosis	ln(A/N)	MI diagnosis	Risk factors	Autopsy findings
ND27760 Coriell	55	F	Parkinson’s disease (PD)	4.6	Non-ADD	N/A	Parkinson’s disease
GM02173 Coriell	52	F	Huntington’s disease (HD)	4.77	Non-ADD	N/A	Huntington’s disease
GM04715 Coriell	40	M	Huntington’s disease (HD)	4.93	Non-ADD	N/A	Huntington’s disease
GM05031 Coriell	60	M	Huntington’s disease (HD)	5.1	Non-ADD	N/A	Huntington’s disease
AG08395 Coriell	85	F	Parkinson’s disease (PD)	5.15	Non-ADD	N/A	Parkinson’s disease, lewy bodies
GM00305 Coriell	56	F	Huntington’s disease (HD)	5.36	Non-ADD	N/A	Huntington’s disease
GM02165 Coriell	67	M	Huntington’s disease (HD)	5.4	Non-ADD	N/A	Huntington’s disease
GM04210 Coriell	59	M	Huntington’s disease (HD)	5.58	Non-ADD	N/A	Huntington’s disease
GM05030 Coriell	56	M	Huntington’s disease (HD)	5.61	Non-ADD	N/A	Huntington disease
GM04222 Coriell	59	M	Huntington’s disease (HD)	5.89	Non-ADD	N/A	Huntington’s disease
GM02167 Coriell	59	F	Huntington’s disease (HD)	5.91	Non-ADD	N/A	Huntington’s disease
ND34265 Coriell	62	M	Parkinson’s disease (PD)	6.01	Non-ADD	N/A	Parkinson’s disease
GM04476 Coriell	57	M	Huntington’s disease (HD)	6.04	Non-ADD	N/A	Huntington’s disease
GM02038 Coriell	22	M	Schizophrenia	6.18	Non-ADD	N/A	Schizophrenia
GM04198 Coriell	63	F	Huntington’s disease (HD)	6.48	Non-ADD	N/A	Huntington’s disease
ND31618 Coriell	58	F	Parkinson’s disease (PD)	6.75	Non-ADD	N/A	Parkinson’s disease
AG06274 Criell	65	F	Huntington’s disease (HD)	6.77	Non-ADD	N/A	Huntington’s disease
564	67	F	Corticobasal degeneration (CBD)	5.8	Non-ADD	Hypertension, hyperlipidemia, osteoporosis, obesity	Corticobasal degeneration, hydrocephalus
574 CRI	90	F	Parkinson’s disease (PD)	5.66	Non-ADD	Macular degeneration, osteoporosis, skin cancer, anemia, transient ischemic attacks, left cerebral artery aneurysm	Parkinson’s disease, multifocal atherosclerosis, arteriosclerosis
586 CRI	69	M	Frontotemporal lobar degeneration with TDP-43-positive inclusions (FTLD-TDP)	6.5	Non-ADD	Hypertension, headaches, cholecystectomy	Frontotemporal lobar degeneration with TDP-43-positive inclusions, hydrocephalus, atherosclerosis of the basilar artery
572 CRI	60	F	Parkinson’s disease (PD), Lewy body disease, neocortical distribution	6.28	Non-ADD	Hypertension, atrial fibrillation	Lewy body dementia, parkinson’s disease, minimal non-occlusive atherosclerosis of circle of willie

Within 30 min to 2 h, skin fibroblasts, when cultured on a 3-D Matrigel matrix, cells come close to forming networks (Fig. 1A, B). The nodes of such networks are cellular aggregates, and the edges are filopodia. The edges start dissociating around 5 h (Fig. 1C). Those dissociated edges leave the nodes and aggerated cellular structures (Fig. 1D, at 24 h and Fig. 1E, at 48 h). In general, AD cell lines formed a smaller number of larger aggregates than non-AD cell lines. Such a difference enabled counting the number of aggregates (N) and measuring aggregates’ average area (A). The quantitative measure, Ln(A/N) at 48 h, is plotted for AD and non-ADD patients from the validation cohorts (Fig. 2). This method accurately diagnosed AD patients and distinguished them from non-ADD (Fig. 2A). The probability distribution of the morphometric imaging signal showed (Fig. 2B) that the AD group (n = 25) separates nicely from the non-ADD group (n = 21). (See Methods section for further details).

**Figure 3:**
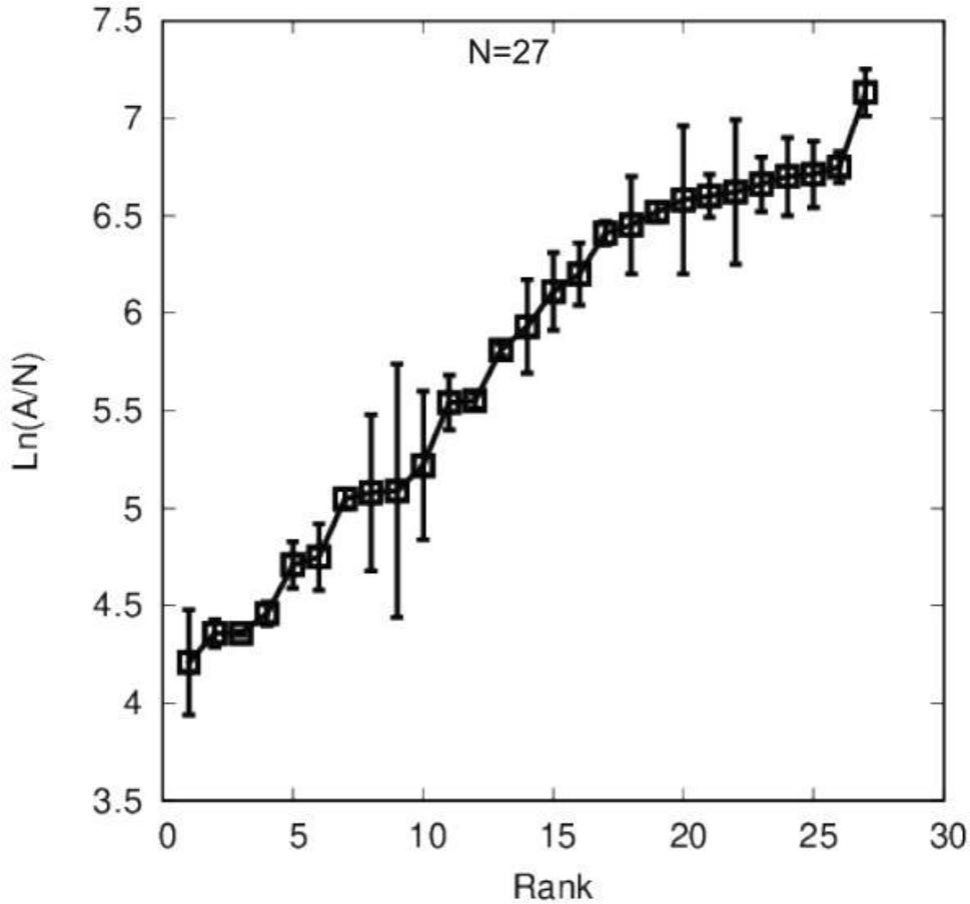
The Apparently Healthy Controls (AHC) ranked in the increasing order of the MI biomarker signal, Ln(A/N), to establish the cut-off in a training study. The population data were ordered according to the Ln(A/N) signal starting with the lowest and ending with the highest value. A linear interpolation was made as X(N) + F * [X(N + 1) + X(N)], where X(N) is the Nth value, X(N + 1) is the N + 1th value, and F is the fractional reminder after taking 0.95 of X(N + 1). Based on this analysis, the cut-off is 6.98.

The Apparently Healthy Controls (AHC) (N = 27) (Table 3; non-autopsy confirmed) were used to establish the cut-off between AD and non-AD cases. AHC ranked in the increasing order of the MI biomarker signal, Ln(A/N), to establish the cut-off during assay development (Fig. 3). The population data were ordered according to the Ln(A/N) signal starting with the lowest and ending with the highest value. A linear interpolation was made as X(N) + F*[X(N + 1)-X(N)], where X(N) is the Nth value, X(N + 1) is the N + 1th value, and F is the fractional reminder after taking 0.95 of X(N + 1). Based on this analysis, the cut-off was 6.98. The basis of the cut-off is to classify AD-specific pathology vs. no AD-related pathology based on values from unaffected controls. The Ln(A/N) value lower than the cut-off value of Ln(A/N) = 6.98 as determined from the biomarker value, was considered an AD diagnosis, and higher than 6.98 corresponds to non-AD; such values might have represented AHC patients or other non-AD dementia patients (if the person had dementia). Application of this cut-off to AD vs. Non-AD patient samples was possible because the distribution of the AHC patient values was superimposable on the Non-AD dementia values^1^.

**Table 3:** Skin fibroblasts-based peripheral diagnosis of healthy control cases (N = 27).

Healthy control cases—Banked coriell samples						
Number	Sample ID	Ln(A/N)	MI Diagnosis	Age	Gender	Original diagnosis
1	AG09977*	4.21	Non-AD	63	F	Healthy control
2	AG11730*	4.36	Non-AD	84	M	Healthy control
3	AG12927*	4.36	Non-AD	66	F	Healthy control
4	AG12998*	4.46	Non-AD	65	M	Healthy control
5	AG04146*	4.75	Non-AD	57	M	Healthy control
6	AG07123*	5.05	Non-AD	62	M	Healthy control
7	AG13358*	5.08	Non-AD	72	F	Healthy control
8	AG04461*	5.09	Non-AD	66	M	Healthy control
9	AG07714*	5.22	Non-AD	56	F	Healthy control
10	AG12438*	5.55	Non-AD	77	M	Healthy control
11	AG05840*	6.66	Non-AD	56	F	Healthy control
12	37**	5.81	Non-AD	65	F	Healthy control
13	39**	7.13	Non-AD	65	F	Healthy control
14	50**	6.70	Non-AD	61	M	Healthy control
15	51**	4.71	Non-AD	55	M	Healthy control
16	19**	6.71	Non-AD	33	M	Healthy control
17	25**	6.05	Non-AD	39	M	Healthy control
18	29**	6.62	Non-AD	21	M	Healthy control
19	32**	6.69	Non-AD	23	M	Healthy control
20	36**	6.41	Non-AD	46	M	Healthy control
21	44**	6.75	Non-AD	50	F	Healthy control
22	73**	6.24	Non-AD	20	F	Healthy control
23	77**	5.81	Non-AD	18	M	Healthy control
24	78**	6.23	Non-AD	45	F	Healthy control
25	82**	6.52	Non-AD	45	F	Healthy control
26	83**	6.58	Non-AD	20	M	Healthy control
27	84**	6.45	Non-AD	21	M	Healthy control

*AD* Alzheimer’s Disease, *EEG* Electroencephalogram, *FAD* Familial AD, *FTD* Frontotemporal dementia, *FTLD* Frontotemporal Lobar degeneration, *LDB* Lewy body dementia, *PD* Parkinson’s Disease, *VaD* Vascular dementia, *CDB* Corticobasal degeneration, *FTLD-TDP* Frontotemporal Lobar Degeneration with TDP-43-Positive Inclusions, *HD* Huntington disease, *PD, LDB* Lewy body disease, *PD* Parkinson Disease, *VaD* Vascular dementia, *Coriell* Coriell Institute, *CRI* Copper Ridge Institute, *UTSW* University of Texas South Western, *WHM* William Hill Manor.

*Banked Coriell Samples;

**Freshly Obtained from the Clinic Alere Samples.

**Figure 4:**
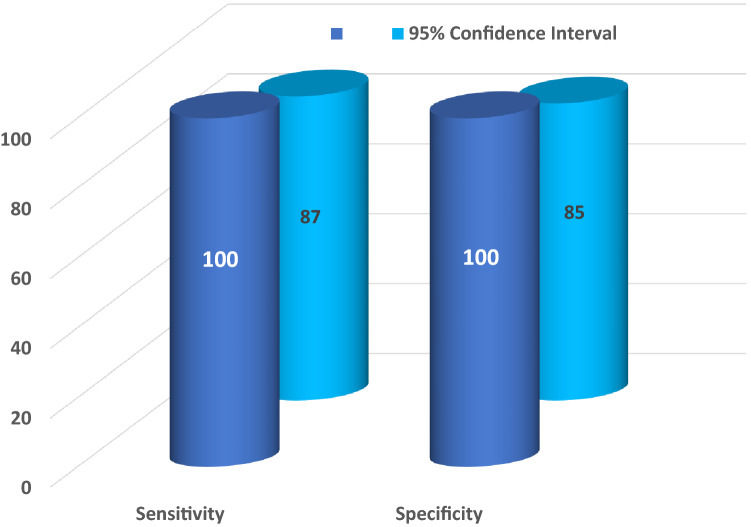
The sensitivity and specificity of the skin fibroblast-based Morphometric Imaging assay and the 95% confidence intervals.

As is apparent in all the tables presented (Tables 1, 2, and 3), the MI assay correctly diagnosed all included patients according to the combined clinical, genetic, and pathologic criteria described above. Furthermore, the MI assay diagnosed AD even for the cases for which AD pathology was present with comorbidities, such as multi-infarct dementia, frontotemporal dementia, Parkinson’s disease, etc. Based on these results, the sensitivity and specificity of the MI assay for diagnosing AD were as follows: sensitivity = 100% (95% confidence interval 86–100%); specificity = 100% (95% confidence interval 84–100%) (Table 4, Fig. 4).

**Table 4:** Diagnoses: MI Imaging Assay versus Autopsy Gold Standard AD (Positive: N = 25) versus Non-ADD (Negative; N = 21).

Statistic	Value (%)	95% CI (%)
Sensitivity	100	86.28 to 100.00
Specificity	100	83.89 to 100.00
Positive predictive value	100	
Negative predictive value	100	
Accuracy	100	92.29 to100.00

https://www.medcalc.org/calc/diagnostic_test.php.

## Discussion

The diagnosis of AD by clinical and currently available laboratory criteria alone has proven challenging and notoriously necessitating validation of a biological marker that would definitively diagnose AD. The classic gold standard autopsy pathologic hallmarks, amyloid plaques, and neurofibrillary tangles appear consistently in both Early-Onset Alzheimer’s disease (EOAD) and Late-Onset Alzheimer’s disease (LOAD) brains at autopsy. Therefore, the EOAD genes provide a genetic profile that identifies AD classic gold standard pathology and thus serves as an autopsy equivalent diagnostic. Validation of an accurate biomarker test to diagnose AD must consistently correlate with dementia in life, plaques, and tangles the “gold standard” criteria regardless of other factors, including comorbidities. Of all AD cases, 3% to 4% can begin early in life—EOAD—and 96% to 97% of cases start later in life, usually beginning after 50 years of age. According to the gold standard criteria, patients who do not have dementia, i.e., Apparently Healthy Controls (AHC-Fig. 3), cannot have AD and thus do not require autopsies to be considered unequivocal validated controls. Because the AHCs are considered unequivocal-validated controls, they are not included in the specificity determinations. In this trial, the AHC patients were used in a training study to provide a “cut-off” or distinguishing line between biomarker values that identify AD and non-ADD patients from the validation study.

When referenced to autopsy-based pathologic, recent technologies being developed to diagnose AD have shown disappointingly low specificity for AD when considering a 95% confidence interval used to evaluate the potential performance with the future patient population. In contrast, the MI biomarker evaluated here showed high diagnostic accuracy when validated with autopsy.

A further difficulty to be met by an accurate AD biomarker test is presented by the frequent occurrence of co-morbid pathologies in the brains of demented patients^15–23^. Furthermore, autopsy studies have demonstrated that many elderly non-demented patients have the pathology of AD and other neuropathological conditions^16,20^ and mild to moderate Alzheimer type dementia with insufficient signature amyloid plaques and tangles^34^. Therefore, the AD biomarker must also identify the AD pathology in the demented patients’ brains, even when the pathologies of other non-AD dementias are also present.

In earlier research^31^, cellular aggregation, measured by LnA/N), was reported for cohorts where only a fraction of the samples was either gold-standard, autopsy-confirmed, or genetically validated. The remaining samples in this study were only clinically diagnosed. In contrast, in the current study, all samples were validated by the gold standard. In addition, the current study aimed to make the number of samples validated by gold-standard autopsy or autopsy equivalent large enough, > 20, to mitigate the risk of misdiagnosing AD. The 95% confidence interval quantifies the risk of AD misdiagnosis via the sensitivity and specificity as a function of the number of AD/Non-ADD patients. With the current numbers per AD and non-ADD cohorts, the lower limit Confidence Interval sensitivity was greater than 86%, and the Confidence Interval for specificity was greater than 84% (Table 4, Fig. 4).

During our testing, we completed a series of experiments utilizing synthetic extracellular matrices (ECMs) from Millipore Sigma and Thermo-Fisher. We suggest that Synthetic ECMs, as opposed to biological alternatives, allow for better control over internal composition and lot-to-lot variation to limit effects on diagnostic accuracy and precision. Initial experiments with alternatives appeared to produce similar results to those seen with Matrigel®. However, additional validation testing will be required to ensure diagnostic reliability.

The objective of the diagnostic technology proposed has been designed to serve as an AD biomarker—as just defined—but with a minimally invasive procedure using peripheral tissue skin fibroblasts. This peripheral tissue approach has recently become possible because accumulating evidence indicates that AD can cause pathophysiological changes in the central nervous system (CNS) and peripheral tissues ubiquitous in the human body^35,36^. Furthermore, several demonstrations that skin fibroblasts can be transformed into neurons via ISPC technology provide additional confirmation of the relevance of peripheral cells to neuronal functions and biology^37–40^. Recently, in hereditary Spastic Paraplegia disease, morphological profiling in peripheral fibroblasts has been used to classify and distinguish between clinical subtypes for drug discovery, and potentially for biomarkers of disease severity and progression^41^.

The CLIA-certified MI assay measures the disease effects in living cells, and its behavior is a way that measures a whole ensemble of defects (e.g., mitochondrial dysfunction, imbalance in kinases/proteases, dysfunction in cellular energy metabolism, cellular senescence, Ca2 + homeostasis, dysfunction in proteasome activity (removal of toxic proteins, e.g., tau, A-beta, etc., and others)^42–45^.

The autopsy-confirmed CLIA-certified AD-Biomarker assay is a surrogate marker in skin fibroblasts. Besides other reasons, one of the key issues for the failure of AD therapeutic trials is incorrect AD patient selection, especially in the early disease state. Clinical confirmation for AD patient selection has only ~ 60% accuracy. Normally used ABeta-related PET incorrectly adds ~ 30% of normal individuals with amyloid plaques. This biomarker technology will be very useful for patient stratification in AD therapeutic trials. Irrespective of AD patients, whether it originated from familial or sporadic nature, the current AD-biomarker will be useful for the detection of AD. Moreover, the assay will unequivocally detect AD pathology in the presence of other non-AD dementia in a co-morbid state. Maybe drugs that rescue increased aggregates possibly rescue Alzheimer’s disease in screening. We might be using this assay in the future for one of the clinical trials currently going on in the same place (ClinicalTrials.gov Identifier: NCT04538066: Bryostatin Treatment of Moderately Severe Alzheimer’s Disease). However, the current biomarker assay might be a surrogate biomarker for AD detection. Furthermore, there are examples available for cancer drug screening when aggregated cancer cells in 3-D Matrigel have been used as tumor models^45,46^.

Fibroblast Extracellular Matrix (ECM) comprises collagen, non-collagenous glycoproteins, and proteoglycans. Matrigel has laminin (~ 60%), collagen IV (~ 30%), entactin (~ 8%), and the heparin sulfate proteoglycan perlecan (~ 2–3%) plus other growth factors (manufacture’s report). Those components are also secreted from cells to create an ECM meshwork that surrounds cells and tissues. The ECM regulates many aspects of cellular function, including the cells’ dynamic behavior, cytoskeletal organization, and intercellular communication. Neurodegenerative disease processes have been shown to involve dysregulation of several relevant proteins, such as α-synuclein (Parkinson’s and Lewy body diseases), tau, and amyloid beta (Aβ) (Alzheimer’s disease) huntingtin (Huntington’s disease), and TDP-43 (Frontotemporal dementia). Several proteins were shown to be expressed both in neuronal cells as well as in skin fibroblasts. For example, α-synuclein, tau, TDP-43, and huntingtin are intracellular proteins, and their aggregates are located in the cytosol or nucleus of neurons, skin fibroblasts, and spread between cells via secretion/uptake of these protein aggregates in the extracellular space followed by re-uptake. Such interactions may be different in AD compared to non-AD cases. Factors that regulate movement and cell adhesion may affect neuronal and peripheral cells, such as skin fibroblasts. PKCɛ plays an important role in fibroblast cell migration^47^. PKC activities are impaired in skin fibroblasts from AD^25^. Skin fibroblast cells from AD patients migrated more slowly in the 3-D matrix downwards. With slower migration, the fibroblasts can form larger aggregates, while control and non-AD dementia cell lines form a smaller but higher number of aggregates. Defective cell signaling molecules such as Ca2 + , diacylglycerol, and arachidonic acid may vary in a disease-specific manner. All those effects may be orchestrated to form larger size but small number of aggregates in AD skin fibroblasts when cultured in a 3-D Matrigel matrix.

## Conclusion

Diagnosing AD by clinical and currently available laboratory criteria alone has proven inaccurate. The study presented here developed a comprehensive autopsy validation of a minimally invasive 3-mm skin AD biomarker that does identify AD, even in the presence of co-morbid neuro-degenerative pathologies demonstrated at autopsy. It is validated with reference to the “gold standard criteria as outlined by an NIH-organized committee: (1.) dementia in life, (2.) presence of amyloid plaque in the brain at autopsy, and (3.) the presence of hyperphosphorylated tau in the brain at autopsy. In the actual method, skin fibroblast cells isolated from commonly available punch biopsies were cultured on a 3-D Matrigel matrix and their movement dynamics were followed by image analysis. AD cell lines formed fewer *larger aggregates* than non-AD cell lines. Such a difference in the morphology of aggregates enabled the counting of the number of aggregates (N) and measuring of the average area (A) of aggregates. The MI AD biomarker, together with two previously described peripheral cell AD biomarkers, was based on their relevance to multiple factors that have been implicated in the etiology of AD. The other two biomarkers are the Protein Kinase C epsilon (PKCƐ)^25^ and the AD-Index assays^2,14^. PKCƐ is an enzyme that regulates synaptic and neural growth and death as well as regulates the formation and degradation of amyloid-beta toxic proteins and tangles. PKCƐ has also been shown to activate all three Abeta-degrading enzymes intrinsic to the brain^48,49^. Skin fibroblast levels of PKCƐ have been shown to correlate with PKCƐ levels in the brain^25^. The AD-Index Biomarker measures the differential expression of Erk1 and Erk2—in response to the natural inflammatory signal, Bradykinin, which is distributed in multiple tissues throughout the body^2,14^. Quantitative imaging of the phosphorylated Erk1 and Erk2 was then used to identify and differentiate AD from Non-ADD and age-matched control specimens^2,14^. The sensitivity and specificity of all three AD biomarkers could derive from the degree to which they encompass a collection of factors such as synaptic loss, neuronal death, inflammation, amyloid deposition, and hyperphosphorylation of tau protein.

## Methods

### Study population

We confirm that all methods were performed according to the relevant guidelines and regulations of the Declaration of Helsinki of Medical Research Involving Human Subjects. Ethical approval was obtained from the Office of Human Subject Research Institutional Review Boards, Johns Hopkins Medicine, Baltimore, MD 21,205; PI: Jason Brandt, Ethical Committee Chair: Dr. Richard Moore.

Patient populations:

1. Autopsy-validated, MI Imaging-based peripheral diagnosis of AD even in the presence of co-morbidity (N = 25) (Table 1);
2. Autopsy/genetically validated skin fibroblasts-based peripheral diagnosis of non-ADD (N = 21) (Table 2); and
3. MI Assay-based peripheral diagnosis of healthy control cases (N = 27) (Table 3).

Clinical sites were in four US locations: UT Southwestern Medical Center in Dallas, Texas, William Hill Manor in Easton, Maryland, Copper Ridge Institute in Sykesville, Maryland, and Marshall University in Huntington, West Virginia, as well as one location National Medical Center in Tottori, Japan. In addition, banked samples were obtained from the Coriell Cell Repository in Camden, New Jersey. According to our inclusion criteria, dementia patients aged 40 to 90 were enrolled, independent of gender, race, or ethnicity. All autopsy-confirmed AD patients, with and without comorbidities of non-ADD are listed in Table 1, all autopsy-confirmed non-ADD patients demonstrated at postmortem are listed in Table 2, and control cases, including banked cells obtained through the Coriell Cell Repository, are listed in Table 3.

### Diagnostic criteria

These criteria were based on clinical diagnoses, genetic identification for familial Alzheimer’s disease (FAD), and autopsy validation for AD and/or non-ADD. Clinical diagnoses were confirmed for all patients who were then further confirmed with genetic analysis or by autopsy criteria for AD and/or non-ADD. Amyloid plaques and neurofibrillary tangles, together with dementia in life, identified AD. The necessary pathologic criteria for other dementias (e.g., multi-infarct dementia, Pick’s disease, and Lewy-body dementia) were cited when AD pathology occurred together with other comorbidities.

### Trial design

Patients (Total N = 73) were first collected within two pre-specified groups: (1) patients with dementia and (2) non-demented control subjects. For the demented patients (MMSE < 27), three parameters were assessed:

1. Clinical diagnosis
2. AD biomarker skin sample—stored in-house in the liquid nitrogen, and
3. Autopsy—conducted at individual sites regulated by Johns Hopkins University.

For the non-demented control subjects (MMSE ≥ 27), parameters (1) and (2) above were assessed. Healthy control patients were obtained from Marshall University in Huntington, West Virginia, and Coriell Cell Repository. Banked skin fibroblasts were directly obtained from the Coriell Cell Repository. We intend to demonstrate that the current biomarker assay is equally effective in diagnosing AD in familial and sporadic patients. The study included all sporadic AD and some familial AD with clinical manifestations of AD with autopsy or genetically validated patients.

### Inclusion/exclusion criteria

Inclusion.

### Dementia group (MMSE < 27)

(i) Age ≥ 40*; (ii) Cognitive decline for at least six months; (iii) Dementia according to DSM-IV; (iv) Probable AD according to NINCDS-ADRDA criteria.

### Non-demented control group (MMSE ≥ 27)

(a) Does not meet criteria for possible or probable AD (b) cognitively normal controls: (c) age-matched (± 5 years group match) (d) no history of cognitive decline.

*Only one Schizophrenia (Non-ADD) patient had age 22 years.

*Exclusion (all groups*) Hemophilia, other bleeding disorders, uncontrolled diabetes (glycosylated hemoglobin > 6.5%), unable to provide skin “punch” biopsy for any reason, cancer. All participants and/or a legal representative signed informed consent for participating under the oversight of a Johns Hopkins University or other local Institutional Review Board (IRB). The autopsy registry and autopsy procedures are described in Supplementary Documents.

### Autopsy registry and procedure

Individuals (or their representatives) at all sites except Marshall University were asked to participate in an autopsy registry so a neuropathological examination of the brain could be conducted upon death. Standard brain preparation and analysis were performed. CERAD criteria were applied to define AD pathology. Additional techniques were used to identify associated pathology, including infarcts, Lewy bodies, or other lesions relevant to dementia. All samples from Marshall University were healthy control cases. Details of the procedures of autopsy diagnosis are available in Supplementary Documents.

### Skin biopsy and cell culture

Skin biopsies (3 mm) from the backside of the upper arm were obtained by skin punch at the clinical sites. Biopsies were placed in a previously sent transport medium, packed into a specialized package, and stored at 2–4 °C. Details of the cell cultures were described elsewhere^14,31^. Our biopsy cell culture team has established skin fibroblast cell lines with a yield of 100%. We tested the effect of the number of passages in our previous study^1^. We found the assay was consistently accurate with passages between 5 and 15. We restricted our assay, therefore, to within these passage limits.

### Important materials used

#### Matrigel

*Supplier* Corning**;** Catalog #: 354,230, Matrigel® Growth Factor Reduced (GFR) Basement Membrane Matrix, LDEV-free; Lot # used: 0,265,001, 0,266,001, 9,077,005, 9,133,005, 1,063,002.

#### FBS

*Supplier* Gemini Bio**;** Catalog #: 100–106, 100–105**;** Lot # used: A57H74L, A90F00I, A23G00J.

#### 12-Well plates

Corning, Costar tissue culture treated plates, Catalog#: 3513 and BD Falcon, Tissue Culture treated plates, Catalog#: 353,043.

### Morphometric imaging (MI) assay

As described previously^30,31^, the 12-well plate coated with Matrigel was removed from the incubator and placed in the biosafety cabinet. Skin fibroblasts in cell suspension adjusted at the cell density of 50 cells/ml were triturated 5 to 10 times with a 5 mL serological pipette to ensure suspension homogeneity. To change the cell density to 50 cells/ml, the number of cells was counted by a hemocytometer. Four wells were seeded by gently pipetting 1.5 mL cell suspension onto the 3-D Matrigel matrix. The target number of cells per 10 × image was 417 (6.033 in natural logarithm format), corresponding to 50 cells/ml initial cell density. We allowed a 3.67% error range in natural logarithm format for the target cell density of ~ 6.033, translating between 5.811 and 6.214. This range in natural logarithmic form translates into 330 to 500 cells/10 × image field; at least five of the nine 10 × image fields for a given well were required to fall within that range to pass.

For the initial cell count, we used a custom ImageJ plug-in in which we ran “DE speckle” three times; we filtered the image three times with a minimum filter of radius 0.5 and then ran “Subtract Background” with a rolling radius of 20. Finally, we made the image binary and ran “Analyze Particles” in the size range 180-Infinity. All these ImageJ commands were run inside a loop so that we could analyze all the images from one cell line automatically in less than two minutes. The ImageJ plug-in was tuned using manual cell counts on the same images, and the relative error was below 7%.

At least three of the four wells were required to pass for a plate to continue the analysis. If two or more wells did not pass, the assay was repeated using a backup T25 flask of cells. Cells on the 3-D matrix were imaged in the attachment phase between 10 and 30 min, depending on how quickly the cells were attached. In the aggregation phase, the cells were imaged for 10 to 30 min to double-check the cell count range with the ImageJ plugin, 3-, 5-, 24- (data not shown), and 48 h post-seeding (Fig. 1).

In the development phase of the assay^30,31^, cells were imaged more often in the attachment and spreading phase. The microscope’s focal plane was adjusted to make the maximum number of aggregates visible at 48 h in each field of view. If any aggregates were not focused well enough to be measurable, an additional image was taken in the appropriate plane to focus them. In the photos at 48 h, aggregate area (A) was measured and averaged, and the number of aggregates (N) was counted. Each image’s unit average aggregate area (A/N) was determined in the natural logarithmic format Ln(A/N). In natural logarithmic format, the unit average aggregate area was averaged over all the qualified images from the well and averaged one more time overall eligible wells. The result of the unit average aggregate area, which we call Ln(A/N), is a measure of cellular aggregation for a patient. Samples from all patients meeting the inclusion and the diagnostic criteria above were evaluated with the MI assay. The tables below provide the diagnostic results of those assay measurements (Tables 1, 2, 3, and Fig. 2).

The fibroblasts used in this study originated from frozen cell stocks grown from biopsies taken at their respective institutions. Each of these lines was also aliquoted and re-frozen after arrival to maintain secondary vials and control overall cell line aging. In order to maintain quality control of lines post-thaw, a series of parameters were defined for cell growth. These parameters include cell/culture viability post-thaw, growth time on a per passage basis, and passage limits for lines in culture. Viability post-thaw was assessed by surveying cell adhesion 24 h after cell seeding. In order to continue culturing and further testing, the flasks would need to show at least 20% confluence after cells were given time to settle (24 h). Growth time limits were assessed on a per passage basis, where any flask that requires more than 14 days to grow to confluence (80–90%) was labeled as a slow grower. Any line or flask that exceeded this limit was labeled as a slow grower, was discarded and ultimately not considered for the study. Regardless of initial dilution prior to seeding, most lines averaged between 4 and 8 days to reach confluence. Lastly, all lines were limited in the number of passages, both in overall age and passage number post-thaw. No line was tested beyond passage 15 (P15) and any flasks that reached that point were replaced with a secondary thaw with a lower passage number. In addition to the overall P15 limit, each line was limited to no more than five subculture events post-thaw, at which the lines were considered to be P + 5. No line was used in experimentation beyond P + 5, and if necessary, a secondary vial would be used to continue any additional investigation. Over the course of the study, AD lines did tend to proliferate at a slower rate than non-Alzheimer’s disease and healthy control lines. However, this difference in rate varied between individual lines regardless of the disease state. In other words, there were limited differences between populations due to a somewhat wide distribution of average growth times. In total, the turnaround time was 6–9 weeks.

The error bars in Fig. 2A represent the standard deviation calculated from the averaged Ln(A/N) score from each plate run. For each plate, 10 images were taken per well, and with four wells run per plate, a total of 40 images were taken. Each completed assay contained between 15 and 36 data points from 40 images which was an average, and, therefore, a standard deviation was calculated using all data points. The points and error bars were the plate average and standard deviation from those calculations. These, by definition, were a collection of technical replicates of the individual image, averaged as an overall plate.

### Process validation with CLIA compliance

We followed the CLIA recommendations in this trial. Any lot or brand change was required to undergo rigorous validation with blinded samples. FBS case, a substitute, was validated in parallel runs on the same samples. CLIA provided an excellent solution to this problem by requiring running positive and negative control samples with unknown samples. For example, we established positive and negative autopsy-confirmed Quality Control (QC) cell lines to test each material. When we found a particular lot of material-qualified QC lines, we booked a lot enough to finish a specific portion of the work. We tested proficiency tests for each operator to qualify to do any tests.

### Statistical analysis

#### Establishment of cut-off value for Ln(A/N)

The cut-off value was used to distinguish between AD and non-ADD samples. The reference interval was determined based on the original 27 healthy control samples, and a cut-off value of 6.98 was calculated (Fig. 3). The cut-off was defined as the 95th percentile for the reference interval for the healthy control subjects (n = 27). The 95th percentile (i.e., the cut-off) established the upper value for the reference interval in normal patients. Based on this approach, the sample population to develop the cut-off was independent of the sample population for the clinical validation analysis using the AD and non-ADD subjects. One individual (individual number 13) in the control group had a value of Ln(A/N) = 7.13. We can expect that, occasionally control case samples will show a relatively high value of AD Biomarker assay^14^. Because some control cases can be closer to the Alzheimer’s pathology (preclinical stage) than others.

### Probability distribution of cellular aggregation signal, Ln(A/N)

For the two groups of AD and non-ADD patients, we binned the values for the natural logarithm of area per number of aggregates, Ln(A/N), into intervals that are inversely proportional to the density of points and fit with Gaussian functions. The AD/non-ADD distribution gap is a buffer zone at four standard deviations.

### Sensitivity and specificity

The diagnostic accuracy of the MI imaging assay against the gold standard autopsy diagnosis is shown in Table 4. 25 out of 25 autopsy-diagnosed AD cases were correctly diagnosed as AD by the MI assay, and all 21 of the non-ADD cases were correctly diagnosed as non-ADD by the MI assay.

## Supplementary Information

Supplementary Information.

## Data Availability

All data will be available on reasonable request from the corresponding author.
