# Supplementary material for: Morphometric imaging biomarker identifies Alzheimer’s disease even among mixed dementia patients
Source: Sci Rep. 2022 Nov 1;12:17675. doi: 10.1038/s41598-022-21796-y (PMC9626495; doi:10.1038/s41598-022-21796-y)
Supplement: Supplementary file 1 — Supplementary Information. [file 41598_2022_21796_MOESM1_ESM.docx]

**Supplementary Document**

***Autopsy diagnosis****.* An autopsy registry was created, and neuropathological examinations conducted upon death. The pathological diagnosis of Alzheimer’s Disease (AD) was performed according to the Consortium to Establish a Registry for Alzheimer’s Disease (CERAD)^1^. These criteria have been the most widely used for the pathological diagnosis of AD in the US and abroad and are based on the semi-quantitative assessment of the density of neuritic plaques on silver-stained sections from various neocortical regions. For clinical-pathological correlation, we used the consensus recommendations for the post-mortem diagnosis of AD from the National Institute on Aging and the Reagan Working Group on diagnostic criteria for the neuropathological assessment of AD^2^. Those recommendations were taken in concert with the CERAD neuritic plaque score and the neurofibrillary Braak score to determine the likelihood (low, intermediate, or high) that Alzheimer’s lesions account for dementia in the patient.

Further details of the autopsy protocol were as follows: During the post-mortem examination, the brain was removed and fixed in 10% buffered formalin. A sample of the frontal pole was frozen and kept at −80^0^C. Following two weeks of fixation, the brain was cut into 1-cm coronal slabs. Tissue samples for histology were taken from the cerebral cortex in each lobe, entorhinal cortex, hippocampus, amygdala, basal ganglia, thalamus, brain stem, and cerebellum. These tissues were processed, embedded in paraffin, and cut at 10mm thickness. Sections were stained with hematoxylin-eosin (H&E) and Hirano silver method, a modification of the Bielchowsky method^3^. Selected sections were immunostained for αε-synuclein for the assessment of Lewy bodies and neurites, and phosphorylated tau to evaluate neurofibrillary changes. Neuritic plaques and neurofibrillary tangles were assessed primarily on silver stains^4^. We used immunostaining for tau to complement the observations with silver stains and to solve occasional questions. Dementia with Lewy bodies was distinguished by examining the substantia nigra and locus coeruleus with H&E stains plus α-synuclein immunostains. Tau immunostains were conducted with tau AT monoclonal antibody with a 1:50 dilution using biotinylated secondary and HRP-conjugated avidin-biotin complex as tertiary. If AD pathology was identified in the autopsy brain, AD diagnosis was considered to have been made with or without the presence of co-existing morbidity due to other neuropathological diseases, such as Parkinson’s disease, dementia with Lewy bodies and so forth.

***Preparation of 3-D Matrigel Matrix***: The methodology of preparation of 3-D Matrigel Matrix has been adapted from previously published studies^5,6^. All materials were kept on ice in the biosafety cabinet and carefully triturated Matrigel with a 1mL micropipette to ensure fluidity – and avoid generating bubbles or froth. 700µl of Matrigel per well was dispensed in each well of the 12-well plate, carefully avoiding any bubble formation. The 12-well plate was incubated at 37 °C in a CO_2_ water jacket incubator for 30 minutes to allow Matrigel to solidify to a gel-like consistency while ensuring the plate was placed level on the shelf. The Matrigel-covered wells were used for the assay within two hours of Matrigel coating.

***Positive and Negative Controls and Cell Density Correction***: Positive and negative controls were matched with unknown double-blinded samples in this trial, as required by the Clinical Laboratory Improvement Amendments (CLIA-1988). Positive and negative controls showed the expected diagnosis of AD and non-AD dementia (non-ADD) respectively for all the experiments. They confirmed the known linear dependence (R^2^>0.95) of the MI signal, Ln(A/N), with the status of the cells before the experiment was measured by cell density. This linear dependence corrects the signal, Ln(A/N), for cell density. Cell density depends on fetal bovine serum (FBS) lot, and the entire trial used the same FBS lot, quality controlled before the trial. We eliminated the FBS lot-to-lot variation by using this correction.

***Variables Controlled in Morphometric Imaging Assay***: We control the lot-to-lot variability of Matrigel used for 3-D matrix preparation by using the same lot emerging from the quality control study before the trial. Matrigel's protein concentration seems to have increased over the years; therefore, we had a quality control study where we tested several lots and picked the best two for the trial. Another variable we tightly controlled is cell seeding, which we double-check with the ImageJ plugin validated with the manual count^5^. The status of the cells before the experiment, intimately related to cell-to-cell interaction, is also a variable we tightly controlled. In addition to the visual confluence of 80% to 90% determined to be optimal, we estimate this confluence via the measured cell density and cell size emerging from the ImageJ plugin^5^.

**Operator-To-Operator Variability for Morphometric Imaging Assay**. Two independent operators tested the same samples to determine the within-laboratory imprecision^6^. One example was below the cut-off and the other example was above the cut-off. There are multiple replicates for the same sample to assess repeatability and trend. These experiments determined that the operator-to-operator variability for Ln(A/N) is within one standard deviation and does not impact the MI diagnoses.

**Biopsy Culture Summary Table with Complete Information of breakdown of the samples from the different centers**: The trial found no effect of center-to-center variation in the fibroblast isolation, and cell growth.

**Table S1: Center-to-center breakdown of biopsy culture summary with complete information**

| **Biopsy Site** | **Country** | **Complete** | **Incomplete** | **Total** | **Transport Days** | **P0-P1 Days** | **P1-P2 Days** | **P2-P3 Days** |
| --- | --- | --- | --- | --- | --- | --- | --- | --- |
| TMC | Japan | 43 | 9 | 52 | 0.0 | 18.3 | 8.7 | 6.3 |
| HCH | Japan | 11 | 1 | 12 | 0.0 | 19.4 | 10.3 | 6.5 |
| UTSW | U.S | 24 | 8 | 32 | 1.0 | 21.0 | 7.1 | 6.6 |
| CRI | U.S | 27 | 9 | 36 | 0.8 | 26.2 | 8.4 | 7.2 |
| WHM | U.S | 9 | 2 | 11 | 1.0 | 23.6 | 7.8 | 7.8 |

CRI, Copper Ridge Institute; UTSW, University of Texas South Western, WHM, William Hill Manor.

| **Overall** | | | | | |  |
| --- | --- | --- | --- | --- | --- | --- |
| **Minimum** | 7.12 | 0 | 13 | 3 | 4 |  |
| **Maximum** | 7.44 | 2 | 50 | 15 | 15 |  |
| **Average** | **7.296** | **0.5** | **21.2** | **8.4** | **6.7** |  |
|  |  |  |  |  |  |  |
| Japanese biopsies were cultured on the same day the biopsies were taken. | | | | | | |
| Most biopsies from the U.S were cultured the day after the biopsies were taken. | | | | | | |
|  |  |  |  |  |  |  |

Only autopsy-confirmed cell lines were included in Tables 1 and 2.

**Supplementary Figures to show the difference between Alzheimer’s disease (AD) and unaffected controls for reference.**


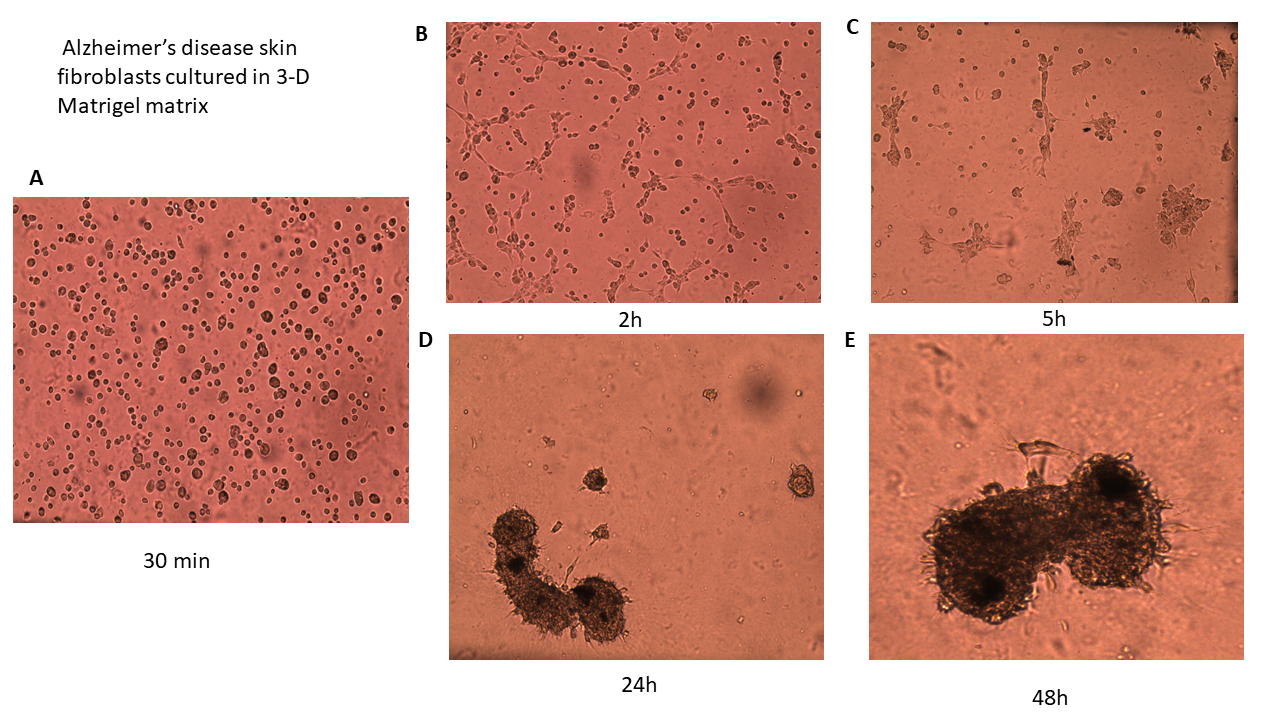


Figure S1: Time course study of cultured skin fibroblasts obtained from Alzheimer’s patients on a 3-D Matrigel matrix. AD and non-ADD dementia cells are cultured, and images are taken at 30 minutes (A), 2-hour (B), 5-hour (C), 24-hour (D), and 48-hour (E) intervals. All images are taken in 10X objective.


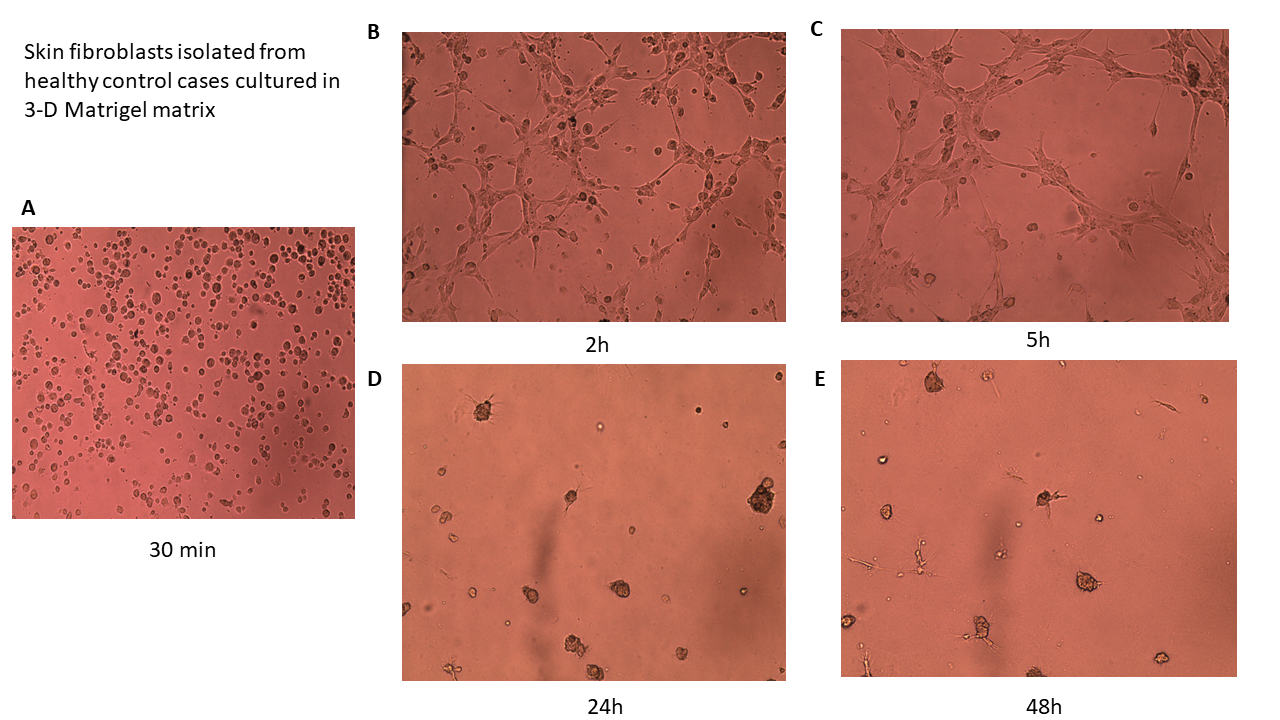


Figure S2: Time course study of cultured skin fibroblasts obtained from unaffected controls for reference on a 3-D Matrigel matrix. AD and non-ADD dementia cells are cultured, and images are taken at 30 minutes (A), 2-hour (B), 5-hour (C), 24-hour (D), and 48-hour (E) intervals. All images are taken in 10X objective.

***Changes in cellular morphologies as a function of time:***

The 48-hour time point was chosen to allow for the complete resolution of networks and complete consolidation of aggregate bodies while also limiting the possibility that cells/aggregates were able to reach the bottom of the well. Images taken at 24 hours showed a similar pattern of aggregation to that of 48 hours; however, at 24 hours, there was a higher likelihood that some networks have yet to resolve. This can lead to less accurate measurements of aggregate size due to a higher number of migrating cells. Images and resulting measurements were not taken beyond 48 hours to avoid the risk of the aggregates falling to the bottom of the well. Aggregations falling on the bottom can result in a secondary migration event where cells begin to spread out of the extracellular matrix and onto the 2-D surface of the plate.

Within 1 to 2 h, skin fibroblasts, when cultured on 3-D Matrigel matrix, cells come into close to form networks (Fig. S3 A, B). Network formation was occasionally well-defined in the case of non-AD cell lines and slow and distorted in AD cell lines. The nodes of such networks are cellular aggregates, and the edges are filopodia. The edges start to dissociate around 5 hours (Fig. S3 C). Those dissociated edges leave the nodes, aggerated cellular structures at 24 hours and 48 hours, respectively (Fig. S3 D, E).


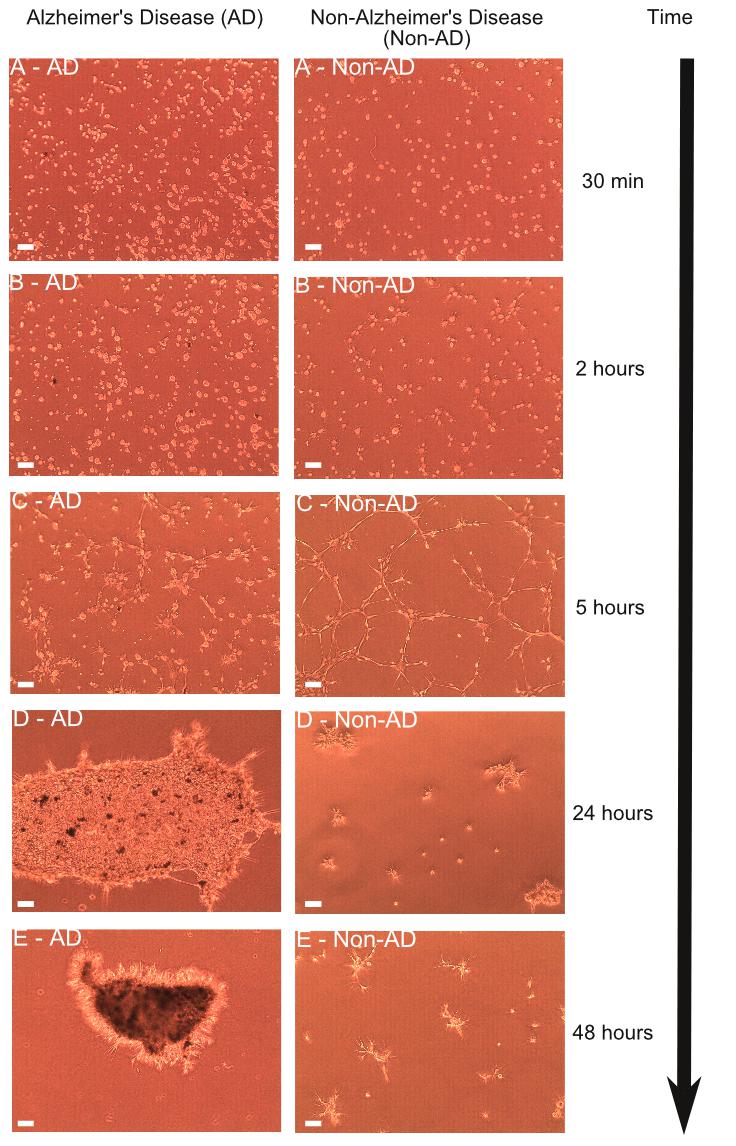


Figure S3: Time course study of cultured skin fibroblasts obtained from Alzheimer’s disease and non-Alzheimer’s disease (AD/non-ADD) patients on a 3-D Matrigel matrix.  AD non-AD dementia cells are cultured, and images are taken at  30 min (A), 2-hour (B), 5-hour (C), 24-hour (D), and 48-hour (E).  Typically, the 24-hour images are not significantly different from the 48 hours.   The "network" - like appearance of the fibroblast aggregates in C, though not frequent, has been seen with non-ADD but not AD samples.  All images were taken with 10X objective. The scale bar is 100 mm.

**References**

1. Mirra SS, Heyman A, McKeel D, Sumi SM, Crain BJ. The Consortium to Establish a Registry for Alzheimer's Disease (CERAD): Part II. Standardization of the neuropathologic assessment of Alzheimer's disease. Neurology, 1991; 41(4):479-86. doi: 10.1212/wnl.41.4.479.
2. Hyman BT, Trojanowski JQ. Consensus recommendations for the postmortem diagnosis of Alzheimer disease from the National Institute on Aging and the Reagan Institu.te Working Group on diagnostic criteria for the neuropathological assessment of Alzheimer disease Journal of Neuropathology and Experimental Neurology 1997; 56:1095-1097. DOI: 10.1097/00005072-199710000-00002.
3. Yamamoto T, Hirano A. A comparative study of modified Bielschowsky, Bodian and thioflavin S stains on Alzheimer's neurofibrillary tangles. Neuropathology and Applied Neurobiology 1986; 12:3-9. DOI: 10.1111/j.1365-2990.1986.tb00677.x.
4. Braak H and Braak E. Neuropathological staging of Alzheimer-related changes. Acta neuropathological 1991; 82 (4):239-259.
5. Chirila FV, Khan TK, Alkon DL. Spatiotemporal complexity of fibroblast networks screens for Alzheimer’s disease. J Alzheimer’s Dis 2013; 33:165-176.
6. Chirila FV, Khan TK, Alkon DL. Fibroblast aggregation rate converges with validated peripheral biomarkers for Alzheimer’s disease. J Alzheimer’s Dis 2014; 42:1279–1294.
